# PTESFinder: a computational method to identify post-transcriptional exon shuffling (PTES) events

**DOI:** 10.1186/s12859-016-0881-4

**Published:** 2016-01-13

**Authors:** Osagie G. Izuogu, Abd A. Alhasan, Hani M. Alafghani, Mauro Santibanez-Koref, David J. Elliot, Michael S. Jackson

**Affiliations:** Institute of Genetic Medicine, Newcastle University, Newcastle Upon Tyne, UK; Security Forces Hostpital, P. O. Box 2748-24268-8541, Makkah, Kingdom of Saudi Arabia

**Keywords:** RNAseq, circRNA, PTES, Splicing, mRNA processing, Software

## Abstract

**Background:**

Transcripts, which have been subject to Post-transcriptional exon shuffling (PTES), have an exon order inconsistent with the underlying genomic sequence. These have been identified in a wide variety of tissues and cell types from many eukaryotes, and are now known to be mostly circular, cytoplasmic, and non-coding. Although there is no uniformly ascribed function, several have been shown to be involved in gene regulation. Accurate identification of these transcripts can, however, be difficult due to artefacts from a wide variety of sources.

**Results:**

Here, we present a computational method, PTESFinder, to identify these transcripts from high throughput RNAseq data. Uniquely, it systematically excludes potential artefacts emanating from pseudogenes, segmental duplications, and template switching, and outputs both PTES and canonical exon junction counts to facilitate comparative analyses. In comparison with four existing methods, PTESFinder achieves highest specificity and comparable sensitivity at a variety of read depths. PTESFinder also identifies between 13 % and 41.6 % more structures, compared to publicly available methods recently used to identify human circular RNAs.

**Conclusions:**

With high sensitivity and specificity, user-adjustable filters that target known sources of false positives, and tailored output to facilitate comparison of transcript levels, PTESFinder will facilitate the discovery and analysis of these poorly understood transcripts.

**Electronic supplementary material:**

The online version of this article (doi:10.1186/s12859-016-0881-4) contains supplementary material, which is available to authorized users.

## Background

Recently, there has been an increased interest in a novel class of transcripts where the exon order differs from that found in the genome [1–4]. Once considered cloning artefacts [5] or products of aberrant splicing [6], it is now established that the majority of these molecules represent circular RNA species (circRNAs) [2–4, 7], although some linear transcripts have been reported [1, 8, 9]. Thousands of these novel transcripts have now been identified in a variety of eukaryotic cells [3, 10], many are conserved across species [2, 11], suggesting functional relevance, and two (from *CDR1* and *SRY*) have been shown to harbour numerous miRNA binding sites and act as miRNA sponges to modulate gene expression [4, 12]. Recent reports also implicate circRNAs in synaptic development [11] and some have expression patterns that correlate with diseases [13–15] and may act as biomarkers for ageing [16]. Despite these reports, the function of the vast majority of these transcripts has not been established.

The defining feature of these transcripts at the sequence level is the presence of a splice junction with exons in an order inconsistent with their position in the genome. As this feature alone does not enable inference of structure or mechanistic origins, we use the term Post-Transcriptional Exon Shuffled (PTES) transcripts to collectively describe this population of RNA molecules [1]. Recent reports have shown that the vast majority of these transcripts emanate from known genes [2, 17, 18], utilise known splice junctions, and that their biogenesis competes with splicing of canonical transcripts [19]. Transcripts arising from PTES specifically exclude chimeric RNAs without known splice junctions, and a class of circular RNAs comprised of spliced introns, ciRNAs [7, 20].

Many computational methods for identifying chimeric RNA molecules from high-throughput RNA sequence data have been described. The majority of these are designed to detect heterotypic trans-splicing and fused genes, so are not suitable for PTES detection [21–27], or require post processing steps to analyse reads supporting different types of splicing events [28, 29]. Recently, however, a number of programs for PTES discovery have been described and used primarily for circRNA characterisation [2–4, 16, 17, 30–32]. Most analyse reads which fail to fully align to a reference sequence, and split these into two or more segments which are then independently aligned to define rearrangements. Some make use of gene annotation to guide discovery [3, 32], while others adopt an unbiased genome-wide approach to capture structures which do not utilise known splice junctions or are non-genic [4, 16, 17, 30, 31]. In addition, the occurrence of PTES can be inferred when two paired end reads map to the transcriptome in a configuration that is not consistent with a linear transcript [3, 16, 30, 32].

**Fig. 1:**
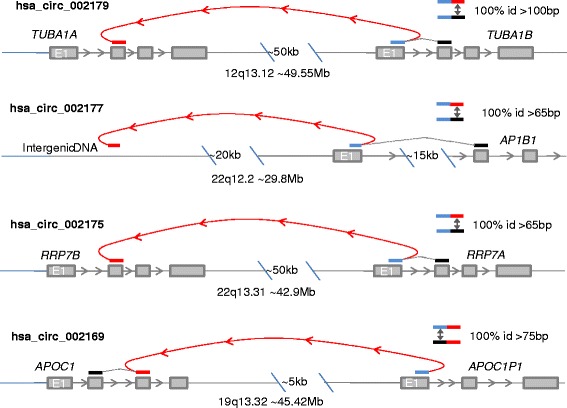
Examples of Intragenic False Positives. Schematic diagrams showing inferred structure and key sequence relationships for 4 of the 20 most abundant circRNAs reported in [3]. In each case, the inferred structure shares 100 % identity to a linear transcript spanning the defining exon-exon junction. Within the top 20, hsa_circ_002174, 002165 and 002164 show similar patterns of identity to multiple genomic locations. *Blue* – Inferred Donor Exon, *Red* – Inferred Acceptor Exon, *Black* – upstream or downstream RefSeq exon sharing 100 % identity to donor or acceptor exon at junction. Approximate chromosomal locations (HG19) are shown

The identification of PTES exon junctions within RNAseq data is, however, confounded by known artefacts. False positives can arise from template switching during cDNA synthesis [1, 5, 9, 33, 34], from genes with duplicated exons [35], from transcription read-through between genes in close proximity due to weak termination signals [36, 37], and from closely related genes within duplicons or tandem arrays [4]. Although experimental enrichment has been combined with informatic approaches to define bona-fide circRNAs [2–4], many classes of false positive structures are not directly excluded by existing identification methods. For example, reads defining 7 of the 20 most abundant human circRNAs reported by Memczak *et al.* [14] map with high sequence identity to the reference sequence and include 4 which are indistinguishable from linear RefSeq entries (Fig. 1). Furthermore, a recent experimental analysis of previously identified PTES trancripts concluded that many are template switching artefacts [38], and template switching predominantly leads to rearragements where the breaks do not occur at splice junctions [5].

Here we present a method, PTESFinder, that identifies putative PTES structures by mapping RNAseq reads to sequence models generated using existing transcript annotation. It then applies a series of mapping and alignment filters to systematically remove known classes of false positives. It does not make use of paired end (PE) mapping information as the lack of intervening sequence precludes such filtering and may affect specificity. We first describe the implementation of this method, and then investigate the effects of different filtering criteria. The program requires certain user adjustable parameters; we therefore also explore systematically the choice of these parameters. Finally, through analysis of real and simulated data, we compare PTESFinder to other publicly available methods [4, 16, 30, 31] which have been used to identify circRNA transcripts in both cell lines and tissues.

## Implementation

### Pipeline for PTES discovery

**Fig. 2:**
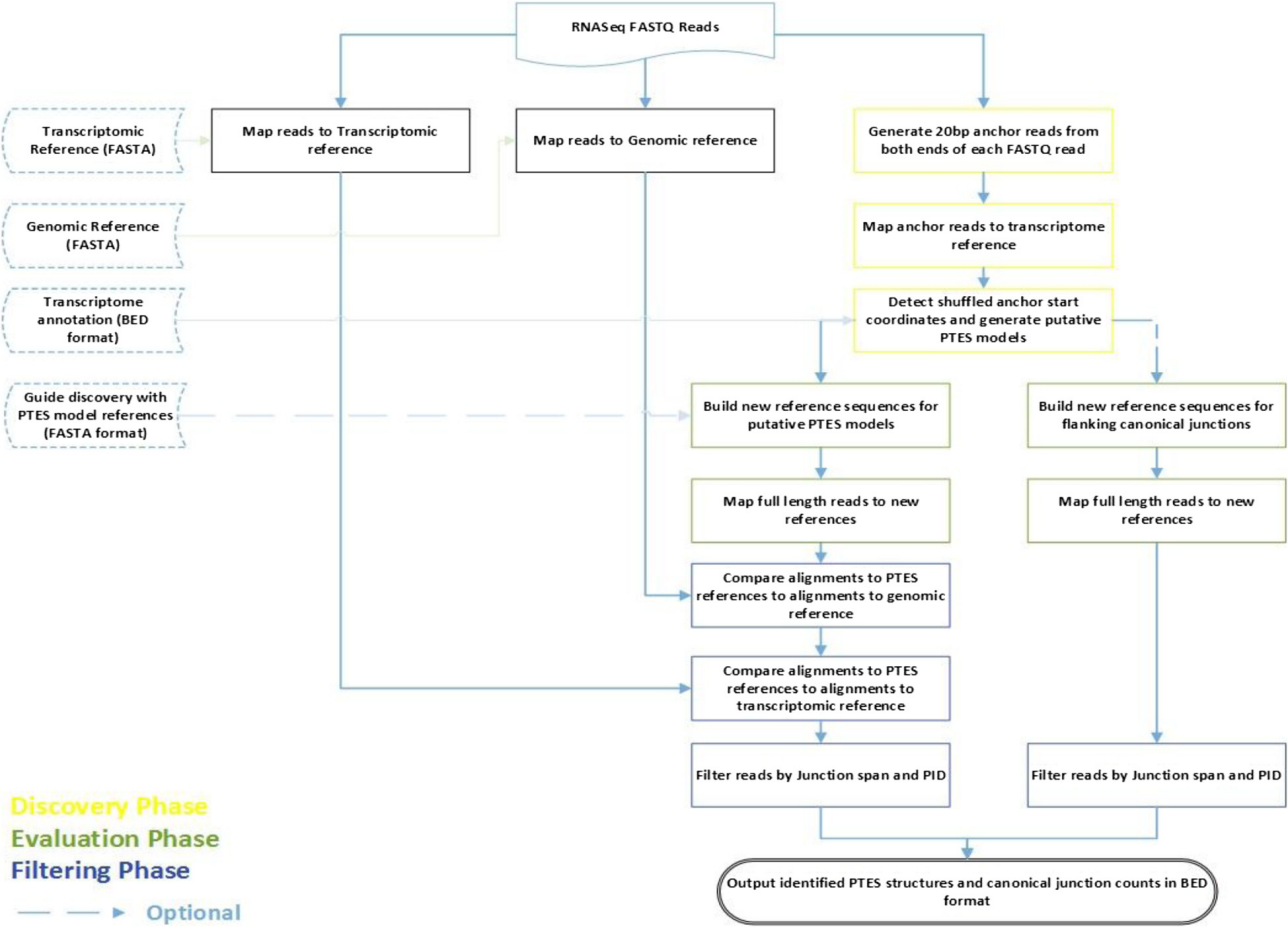
Overview of PTES Discovery Pipeline. The workflow includes three major phases: Discovery phase, Evaluation phase and Filtering phase. Putative PTES structures discovered using 20 bp anchor reads are evaluated by aligning full FASTQ reads to the models. The filtering phase includes stringent criteria designed to systematically exclude all known classes of false positive structures

PTESFinder requires as input files: RNAseq data in FASTQ format [39], genomic reference in FASTA format, and an annotated transcriptome reference in BED format [40]. The pipeline is split into three phases (Fig. 2): A discovery phase to identify putative PTES structures within RNAseq data and define PTES transcript models, an evaluation phase to assess these PTES models, and a filtering phase to exclude potential false positives.

#### Discovery phase

Short sequences from both ends of each FASTQ read (anchors, default length: 20 bp) are aligned to the transcriptome reference using Bowtie [41] with tolerance for a single mismatch. Pairs of anchors from the same read that map to the same gene and in the same orientation, but which map in inverted order with respect to their order in the sequencing read, are then identified. This excludes reads emanating from fused genes and sense-antisense template switching artefacts. Retained anchor pairs are then used to determine the exon junctions which define putative PTES events and create sequence models (constructs) of the inferred products. These constructs are generated by concatenating the last 65 bp of the 5′ exon and the first 65 bp of the 3′ exon. The segment size of 65 is used by default, with the full exon sequence used if an exon is smaller than 65 bp. This parameter is adjustable to accommodate various RNAseq read lengths, and we recommend that it be at least 10 bp shorter than the read length to ensure that only reads mapping across PTES defining junctions are processed in subsequent filtering steps (although as described below these filters would eliminate such reads).

#### Evaluation phase

All the original reads are then re-mapped to PTES constructs generated in the discovery phase. This serves two purposes. First, as RNAseq reads can be short, this enables reads with putative PTES exon junctions close to their termini to be accurately mapped. Second, it enables read mapping scores obtained using the PTES constructs to be directly compared to scores obtained from genomic and transcriptomic alignments for filtering purposes (see below). Optionally, evaluation can also be ‘guided’ by supplying constructs of previously discovered PTES structures, effectively bypassing the discovery phase.

#### Filtering phase

To eliminate potential false positives originating from the genome under investigation, all the original reads are mapped to both genomic and transcriptomic references. The number of edits required for alignment (NM field in SAM format [42]), and the number of perfectly aligned base pairs, are used to remove reads which align as well or better to either of these reference sequences than to the PTES constructs. To reduce template switching artefacts, which have heterogeneous junction points within short regions of often imperfect sequence homology [5], reads which do not align perfectly to the exon junctions which define PTES are also removed using junctional filters. First, a user adjustable minimum junction span (JSpan) parameter is applied to ensure that there are no mismatches or indels within ‘n’ nucleotides either side of the junction position, where n is an even integer. Second, to eliminate reads with regions of low quality alignment, a user adjustable segment percent identity (PID) parameter is also applied independently to the segments on either side of the PTES junction, such that for a read to be retained both must meet or exceed the specified PID when aligned to the PTES construct. These user adjustable filters rely on alignment summaries provided by the NM field, MD field and Cigar in the SAM files [42]. The output includes the coordinates of the exon end involved in the junctions, a descriptor of the PTES (see Additional file 1 for details) and the number of reads supporting the structure. This is presented in BED format [40]. A second file contains additional information, read counts of all canonical exon junctions from transcripts where a PTES structure has been identified, to facilitate comparison with PTES counts.

### Assessment of pipeline and comparisons to other methods

RNAseq data from Jeck *et al.* [2] were analysed at various combinations of JSpan and PID (JSpan range: 4–14; PID range: 60–100 %). All analyses were performed with and without genomic and transcriptomic filters applied to enable reads discarded by each filtering criterion to be identified. The numbers of PTES structures identified and supporting reads were also recorded. To assess sensitivity and specificity, simulated datasets were generated using all published PTES structures within circbase.org [43]. For each dataset, 5000 PTES junctions were randomly selected along with 5000 canonical junctions, and constructs were generated for each junction by concatenating the full sequence of both exons involved in each case. 100 bp simulated reads with random start positions within each construct were then generated. Scripts published by Memczak *et al.* [14] (default parameter values), CIRI v. 1.2 [30] (default parameter values), circRNA_finder [16] (default parameter values), and MapSplice v. 2.1.5 [31] used in [2] (parameters: −-fusion --non-canonical -p16), were compared to PTESFinder by analysing leukocytes cell line RNAseq data (described in [3, 4]), fibroblasts RNAseq data (described in [2]), and simulated data. For each simulation, transcripts correctly identified by each method were determined by comparing genomic coordinates of identified transcripts with the genomic coordinates of transcripts expected to be recovered from within each dataset. The numbers of correctly identified PTES transcripts (true positives – TP), incorrectly identified PTES transcripts (false positives – FP), PTES transcripts incorrectly excluded (false negatives – FN), and canonical junctions correctly excluded (true negatives – TN), were used to estimate sensitivity: TP / (TP + FN), specificity: TN / (TN + FP), and false discovery rate (FDR): FP / (TP + FP).

## Results and discussion

**Fig. 3:**
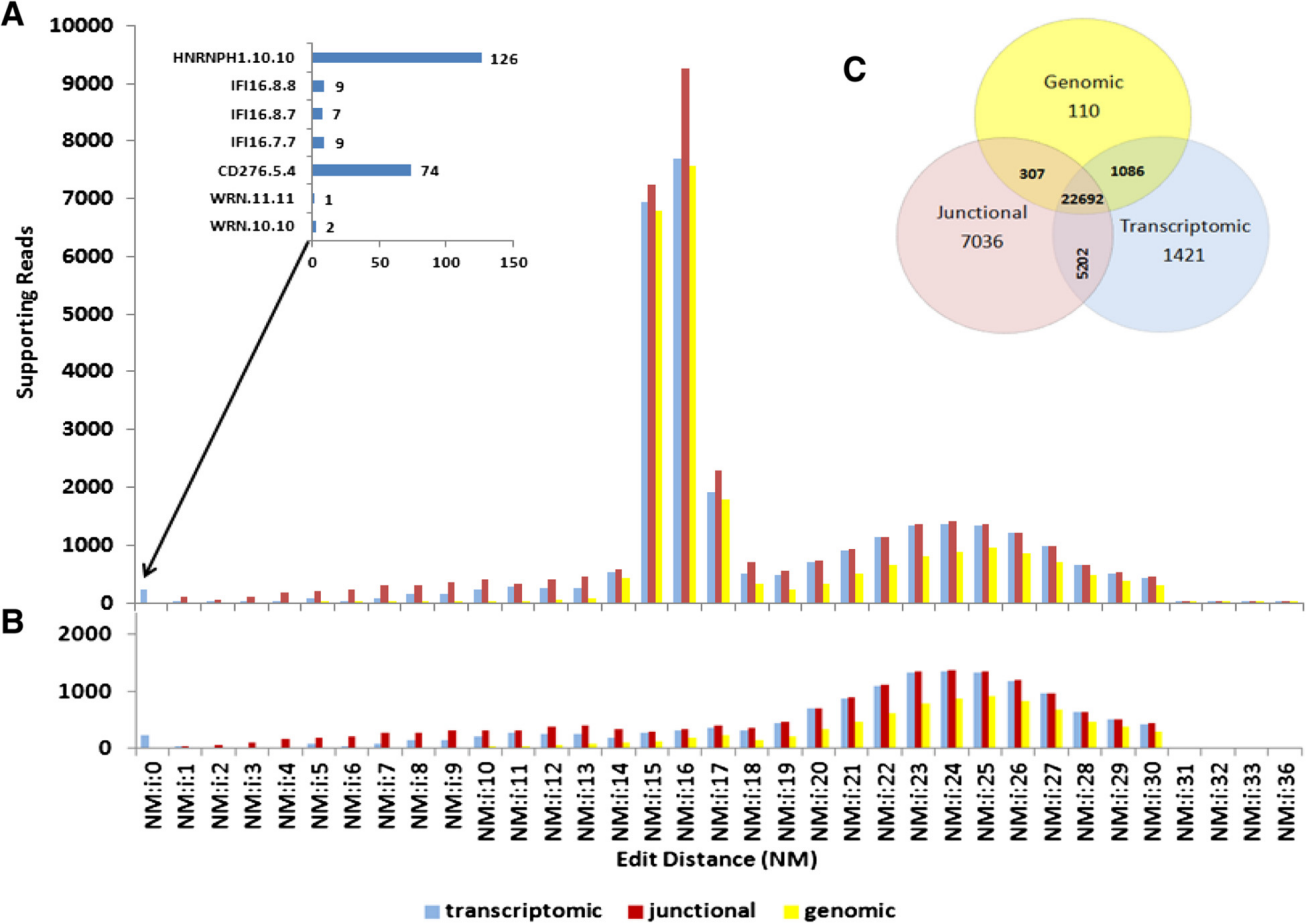
Summary of Reads Excluded by Filters. **a** Edit distance distribution of reads filtered out by genomic, transcriptomic and junctional (JSpan/PID) filters. Inset: Seven structures are supported by 228 reads with 100 % alignment but are excluded by the transcriptomic filter. **b** 30 % of reads filtered out support a false positive structure from 5.8 s rRNA and are excluded in this plot. **c** Venn diagram showing number of reads excluded by filters. Majority of false positive reads are excluded by all three filters. Each filter also excludes a distinct population of false positive reads

PTESFinder uses established RNAseq tools (Bowtie [41], Bowtie2 [44] and Bedtools [45]) to identify putative PTES structures, and then systematically excludes known classes of false positive structures by applying genomic, transcriptomic and junctional (JSpan & PID) filters (see methods). As an initial assessment of PTESFinder function, RNAseq data from human fibroblast total RNA which has previously been mined for circRNAs (sample SRR44975A in [2]), were analysed both with and without the application of the genomic and transcriptomic alignment filters. Reads recovered during analysis, together with alignment edit distances of reads identified by each filter applied seperately, are shown in Fig. 3a.

### Filters target overlapping populations of reads but none is redundant

From a total of over 200 million reads analysed, approximately 0.17 % (359837) have shuffled coordinates with respect to exon position (Fig. 3c and Additional file 2: Table S1). However, of these only 44620 (~12.5 %) map to PTES sequence constructs generated during the discovery phase, indicating that most of the reads with rearranged anchor pairs do not map to single genes and/or known exon junctions. Approximately 85 % (37854) of the reads which map to PTES constructs are subsequently removed by the genomic, transcriptomic and junctional (JSpan and PID) filters, with the majority being identified by more than one filter. For instance, over 98 % of reads excluded by the genomic filter are also excluded by the transcriptomic filter, and 60 % (22692) of all filtered reads are identified by all three. Most of these have high edit distances (>10) indicative of low quality alignment. Despite this, the genomic, transcriptomic and junctional filters (at lowest stringency) uniquely exclude ~0.25 % (110), ~3.2 %% (1421) and 15.8 % (7036) of reads mapping to PTES models respectively (Fig. 3a), indicating that none is wholly redundant.

The subset of reads identified specifically by the junctional and transcriptomic filters are defined by low edit distances of between 1 and 10 (Fig. 3a), although a small number of reads excluded by the transcriptome filter (228) map perfectly to putative PTES constructs with NM = 0 (inset). Fig. 3a also reveals a bimodal distribution of mapping qualities for reads excluded by all three filters with peaks at NM = 16 and NM = 24. Upon manual analysis, most of the excluded reads with NM = 16 were found to support a false positive structure from 5.8 s rRNA (NR_003285.1.1). Comparable rRNA derived structures have been identified previously and filtered manually [4]. In Fig. 3b, reads supporting this structure have been removed to show the underlying distribution of mapping quality scores.

### Reads excluded by specific filters have different origins

To investigate the activity of specific filters further, the mapping co-ordinates of reads removed by the genomic filter were first compared to the co-ordinates of annotated pseudogenes and segmental duplications. This established that ~74 % of reads excluded by the genomic filter had superior alignments to segmental duplications, and ~12 % had superior alignments to pseudogenes. The 417 reads identified by the genomic filter but not by the transcriptomic filter were also found to be enriched for reads derived from segmental duplications and pseudogenes (e.g. Additional file 3: Figure S1A).

We next used BLAT [46] to manually investigate the 228 reads excluded specifically by the transcriptome filter which mapped perfectly to putative PTES constructs (NM = 0, Fig. 3b). These support 7 putative PTES structures from 4 genes (Inset, Fig. 3a). However, BLAT analysis established that they all also mapped contiguously with ~100 % identity to the transcriptome due to high sequence identity between neighbouring exons. For example, 126 reads which support a putative single exon PTES structure (exon 10 of *HNRNPH1* circularized) map with ~100 % identity to exons 10 and 11 of the canonical *HNRNPH1* transcript (Additional file 3: Figure S1B) due to high sequence identity between these neighbouring exons. As a result, these reads cannot be taken as supporting evidence for PTES. It is noteworthy that such structures will pass any qualitative filter criterion requiring only unambiguous mapping to PTES constructs, illustrating the value of the transcriptome filter.

Finally, manual analysis of a subset of the 7036 reads identified only by the junctional filters established that these support structures with distinct patterns of suboptimal mapping, such as low alignment quality specific to only one of the two exons in the structure (e.g. Additional file 3: Figure S1C top 2 panels), and low sequence identity specifically at the junction (e.g. Additional file 3: Figure S1C lower 2 panels), the latter being consistent with the expected pattern of alignment for template switching artefacts [5].

As one further assessment of the filters, we analysed RNAseq data derived from fibroblast RNA which had been pre-digested with RNase R. This selectively removes linear RNAs, and enriches for circRNAs [7, 47], and has been shown to significanty increase the recovery of PTES reads. However, we would anticipate that this would also selectively remove false positives derived from pseudogenes and segmental duplications which mimic PTES structures, without necessarily reducing reverse transcription artefacts such as template switching. Only ~12 % of reads from the RNAseR digested sample which map to PTES sequence constructs are identified by the genomic and transcriptomic filters (Additional file 2: Table S1), compared to 69 % in the undigested sample. Furthermore, only 17 % of these map to segmental duplications, compared to 74 % in the undigested sample. In contrast, the proportion of reads excluded by the junctional filters is considerably higher after RNAseR digestion, consistent with expectation.

### PID Has greater impact than JSpan

**Fig. 4:**
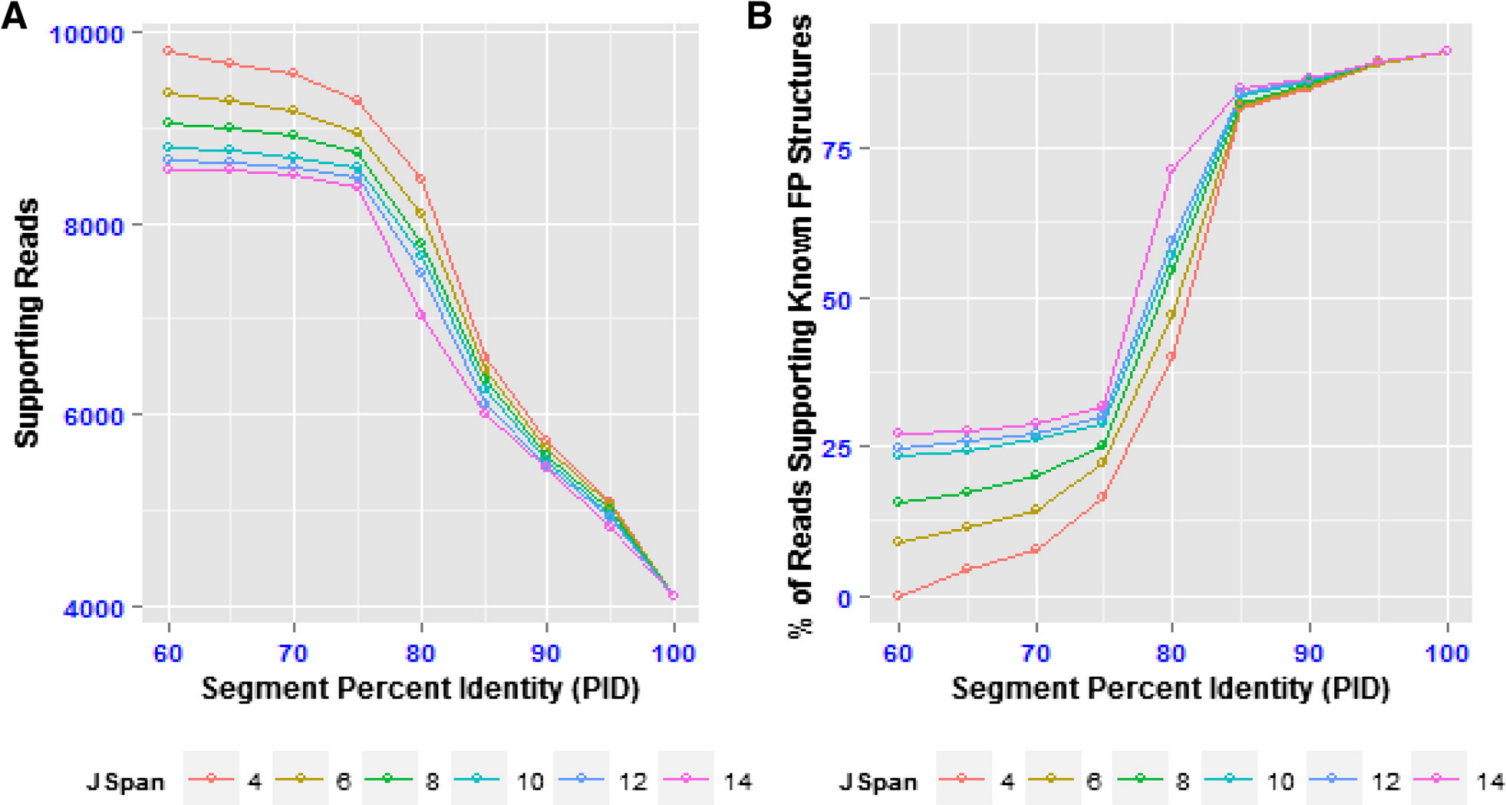
Effect of varying junctional filter parameters. **a** Number of reads passing filter at different combinations of JSpan and PID. **b** Percentage of reads only excluded by transcriptome and genomic filters at default settings, which are filtered at different combinations of JSpan and PID

To investigate the impact of varying the user defined JSpan and PID parameters which comprise the junctional filter, the same data was re-analysed using 54 different combinations of these parameters, both with and without the genomic and transcriptome filters applied (Fig. 3c). This established that varying the PID has a greater impact than varying the JSpan, with 5691 reads filtered with maximal PID (100 %) and lowest JSpan (4) compared to only 1235 reads filtered with the maximal JSpan (14) at lowest PID (60 %). Furthermore, varying the PID between 60 % and 75 % has little impact at any JSpan value, but above 75 % there is a linear relationship with the number of reads filtered. As the default junctional filter parameters failed to identify some reads excluded by the other filters (110 and 1421, Fig. 4a), this analysis was repeated using only these reads to establish the JSpan and PID parameters required to identify them. Over 99 % of these reads are excluded with the most stringent junctional filter parameters (Fig. 4b). Furthermore, the vast majority are filtered with a PID of 85 %, suggesting this is a logical setting for this parameter. The JSpan setting only has a major impact at low PIDs (60–75 %).

### Specificity, sensitivity and comparison with other methods

To assess the sensitivity and specificity of the pipeline and compare it to other methods, simulated reads were generated from previously identified PTES and associated canonical transcripts, and analysed at various read depths of coverage using default parameters. In addition to assessing PTESFinder for *de novo* PTES discovery, the use of constructs of previously reported structures for guided discovery was also assessed (see methods), as were four publicly available methods which have previously been employed to identify circRNA transcripts: MapSplice v 2.1.5 [31] used in [2], CIRI v. 1.2 [30], circRNA_finder [16] and the method used by Memczak *et al.* [4].

**Fig. 5:**
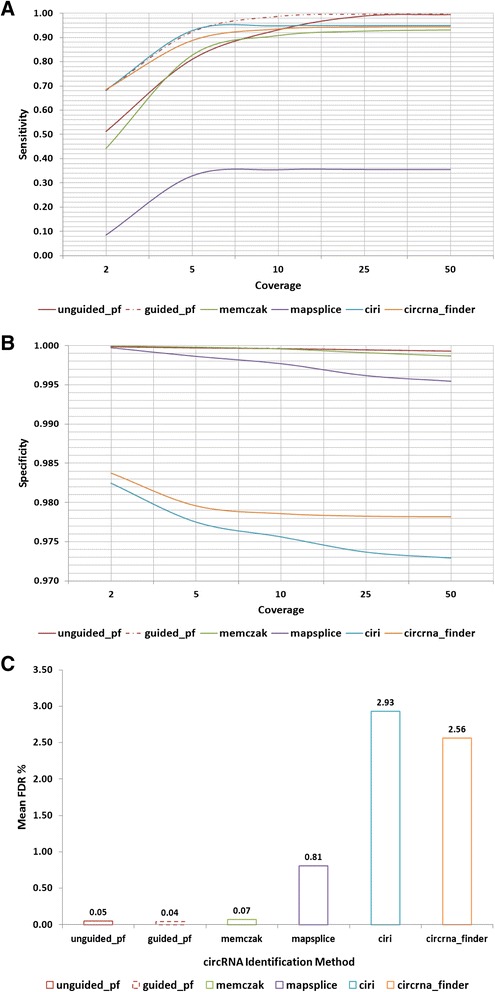
Sensitivity and Specificity in Comparisons to Other Methods. **a** Sensitivity and (**b**) Specificity of PTESFinder and 4 other publicly available methods (CIRI, circRNA_finder, MapSplice and Memczak) analysed using simulated data (see methods). **c** Mean false discovery rate % (FDR) of all methods averaged across all read depths analysed

Results from 100 simulated datasets are presented in Fig. 5a-c, and illustrate that sensitivity varies considerably with coverage, and between methods. At read coverage of 2, the sensitivity of PTESFinder is below 0.6. This can be attributed to PTES junctions occurring within the terminal 20 bp of reads, as the low tolerance for mismatches during anchor mapping will result in their elimination. However, sensitivity reaches >90 % at coverage of 10 or higher for both guided and unguided analyses, with guided PTESFinder being equally or more sensitive than all other methods at all read depths. Strikingly, the sensitivity of MapSplice is low, remaining below 0.5 at all read depths. In contrast, specificity is over 0.97 for all methods at all read depths (Fig. 5b), although PTESFinder achieves the highest specificities averaged across all depths (over 0.999) for both *de novo* and guided PTES discovery, with all canonical junction reads being correctly identified as such within the simulated data. Only the Memczak method has similar specificity when averaged across all read depths (Fig. 5c).

To compare performance using real data we first reanalysed data from Jeck et al. [2] using all 5 methods (Table 1). To allow direct comparison to PTESFinder, the number of putative circRNA structures identified which utilise 2 RefSeq splice sites was recorded for all other methods (bracketed), as the total numbers include structures from intergenic and intronic regions of the genome. For all 4 samples analysed, CIRI consumed >90Gb of memory, resulting in incomplete analyses. It was therefore not analysed further. Of the remaining 4 methods, PTESFinder identified on average 15 % more structures than the Memczak method and ~70 % more than MapSplice. The latter is consistent with our finding that MapSplice, which was used in their analysis [2], has low sensitivity at all depths of sequence coverage (Fig. 5a). However, circRNA_finder reported the highest number of putative circRNA transcripts from both exonic and non-exonic regions of the genome, reporting approximately 31–42 % more structures with RefSeq co-ordinates than PTESfinder (Table 1).

**Table 1:** Number of PTES transcripts identified from Human Fibroblast samples using four methods

Method		SRR444974	SRR445016	SRR444975	SRR444655
Memczak^a^	Identified	22663 (17752)	22351 (17231)	3733 (2956)	1667 (873)
	Run Time	1993 m	2479 m	2602 m	2061 m
MapSplice^a^	Identified	9701 (7087)	7380 (4891)	2231 (986)	1479 (307)
	Run Time	6167 m	16356 m	7412 m	2605 m
PTESFinder	Identified	25116	24489	5383	2316
	Run Time	1355 m	1963 m	1530 m	1369 m
circRNA_finder^a^	Identified	49901 (32856)	54154 (32186)	11069 (7309)	3130 (2131)
	Run Time	75 m	90 m	80 m	88 m

^a^circRNAs utilizing two RefSeq annotated splice sites in brackets

**Fig. 6:**
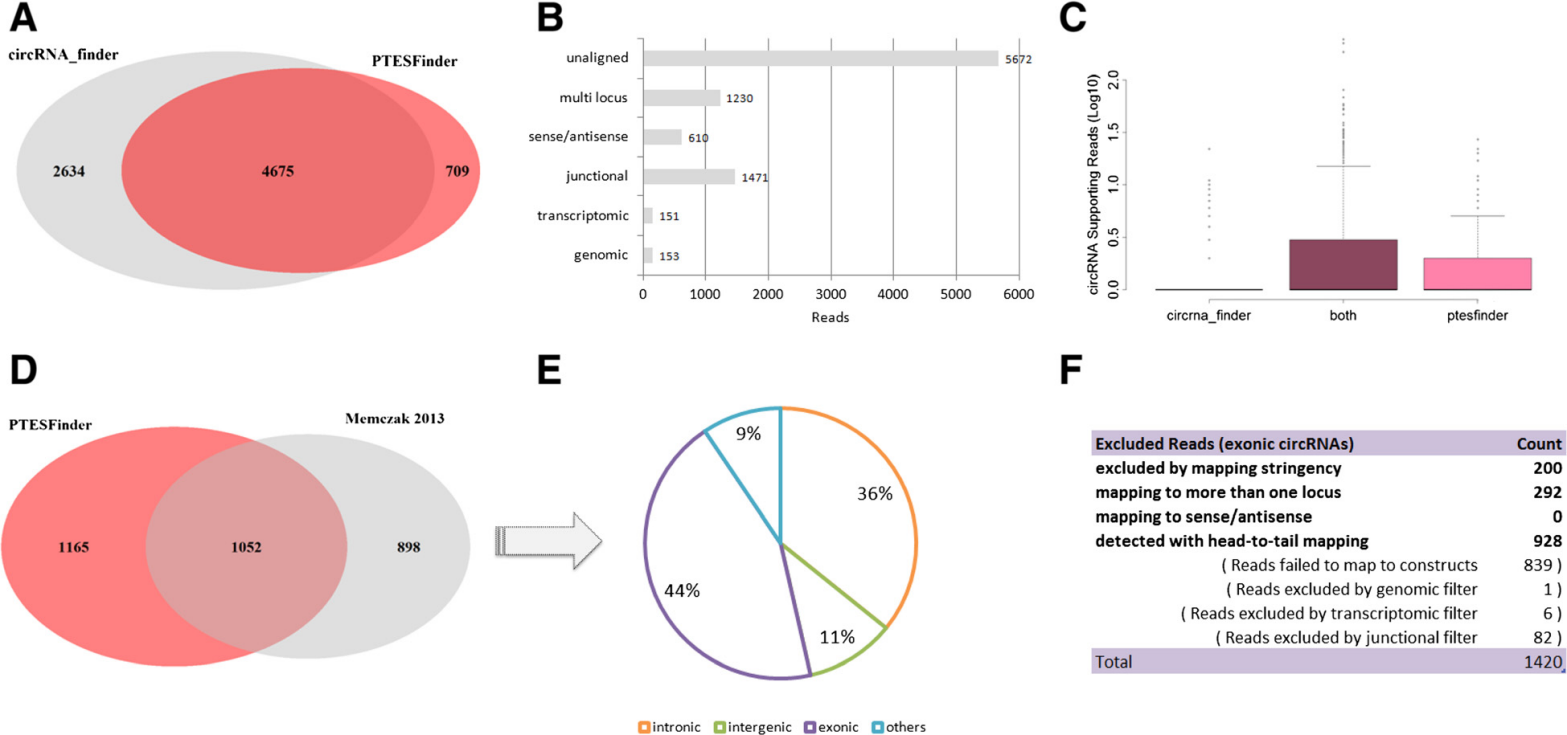
Comparisons with real RNAseq data & published results. **a** Approximately 64 % (4675) of PTES transcripts utilising 2 RefSeq (known) splice sites were identified by both circRNA_finder and PTESFinder from SRR444975 (**b**) Read exclusion criteria for PTES transcripts identified by circRNA_finder only, when analysed by PTESFinder (**c**) Distribution of read numbers supporting PTES transcripts identified by circRNA_finder only, by PTESFinder only, and by both (raw counts reported by PTESFinder shown) (**d**) PTESFinder identified over 50 % (1052) of transcripts reported in Memczak *et al*. [14]. **e** The majority of the 898 structures reported by Memczak *et al.* [14] but not identified by PTESFinder are intronic or intergenic. **f** Exclusion criteria for reads presented as evidence for exonic structures in Memczak *et al*. [14] which were not reported by PTESFinder (see text)

To investigate the origins of the RefSeq related structures identified exclusively by circRNA_finder, reads defining these structures from 1 sample (SRR444975) were re-analysed using PTESFinder (Fig. 6a). Of 9287 reads re-analysed, approximately 20 % (1840) are defined as mutilocus or sense-antisense fusions, and a further 19 % (1775) are eliminated by the junctional, genomic, and transcriptomic filters indicating likely false positives (Fig. 6b). The remaining 61 % (5672) are not aligned, indicating that their anchors map suboptimally to RefSeq. Furthermore, plotting the distribution of the number of reads supporting each structure identified by circRNA_finder only, by PTESFinder only, and by both methods (Fig. 6c), revealed that the vast majority of structures identified by circRNA_finder alone are supported by a single read. This is in sharp contrast to structures identified by both methods, or by PTESFinder alone. While these single-read structures may include *bona fide* low frequency circRNAs, they are also likely to contain false positives caused by suboptimal mapping, consistent with the lower specificity of circRNA_finder with our simulated data.

Runtimes for PTESFinder were also 25–35 % lower than for the Memczak method, and 50–82 % lower than for MapSplice (Table 1), but by far the best runtimes were achieved by circRNA_finder which utilises the STAR aligner [48]. These were, however, achieved at higher computing memory cost (~30GB).

We then used PTESFinder to analyse RNAseq reads previously mined in two further studies [3, 4]. Consistent with the above, it identified 13 % more distinct structures from leukocyte and HEK293 data than were reported by Memczak *et al*. [4] (2217 as opposed to 1950 Fig. 6d), and 41.6 % more structures than reported by Salzman *et al*. [3] from leukocyte data (1875 as opposed to 1324, (data not shown)). As both structures and supporting reads were reported by [4], it was possible to re-analyse the 898 structures identified exclusively by their method using PTESFinder. This established that none correspond to structures which PTESFinder is designed to identify (Fig. 6e): 503 (56 %) are derived from intronic, and intergenic regions, and of the 1420 reads supporting the remaining 395 genic structures, 492 were excluded by PTESFinder due to low map quality (200) or multiple map locations (292), 89 reads were excluded by PTESFinder filters, and the remaining 839 possessed at least 1 exon boundary which did not map to known splice junctions (Fig. 6f). Again, while some of these latter reads will undoubtedly correspond to *bona fide* PTES structures (as a number of genic PTES utilising non-Refseq splice sites have been confirmed experimentally (e.g. [1, 4]), further BLAT analysis established that 13 mapped in a linear fashion to 6 annotated pseudogenes (Additional file 4: Table S2).

Approaches to PTES discovery involve a compromise between the ability to detect all potentially rearranged transcripts, and the ability to identify artefacts generated as a result of the sequence and structural complexity of eukaryotic genomes, and of current library construction methods. It is now clear that the majority of transcripts with re-arranged exon order utilize known exon junctions [2, 18] which are processed by the spliceosome [17, 19]. As a result, methods which utilise existing transcript annotation from the genome under study, such as PTESFinder and those employed by [3, 32], benefit from the reduced noise inherent in this approach and are suited to quantitative analyses of PTES structures that can be characterized using existing annotations.

The use of known/experimentally verified splice sites does reduce the misidentification of template switching artefacts or unspliced pseudogenes as bona fide PTES transcripts. However, it does mean that not all rearranged transcripts will be identified. Although a recent analysis of human data unconstrained by existing annotation suggests that circRNAs which function as miRNA sponges are rare [17], discovery of transcripts which do not utilise known splice sites (including any which are not processed by the spliceosome) requires a genome-wide approach unconstrained by existing annotations. Such approaches are, however, inherently more susceptible to artefacts. The analyses presented above illustrate both the problem of false positive structures, the trade off between sensitivity and specificity in all methods designed to identify rearranged transcripts, and the utility of multiple filters designed to target distinct populations of known artefacts.

## Conclusions

A major challenge in PTES identification is to discriminate between bona fide PTES structures and a wide variety of false positives with distinct origins. Currently, no method which has been used for PTES discovery explicitly excludes all known classes of false positive reads. To that end, we have developed PTESFinder to identify both linear and circular PTES transcripts from high throughput RNAseq data. Compared to publicly available methods recently used in circRNA discovery, PTESFinder achieves higher specificity and sensitivity, and generates output tailored for downstream comparative analyses of transcript abundance, making it an appropriate tool to investigate these RNAs within complex mammalian genomes.

## Availability and requirements

- **Project name:** PTESFinder
- **Project home page:**http://ptesfinder-v1.sourceforge.net/
- **Operating system(s):** Linux
- **Programming language:** Shell, Java 1.6
- **Other requirements:** Bowtie 1.1.1 & 2.2.4, BedTools 2.22.0
- **License:** OSI-Approved Open Source (Artistic License 2.0)
- **Any restrictions to use by non-academics:** None

## Additional files

Additional file 1:
**Distinct PTES structures identified from dataset mined in Memczak et al.** [4]. (XLSX 1443 kb) Additional file 2: Table S1. Analyses of RNASEQ data from human fibroblast cells. (PDF 19 kb) Additional file 3: Figure S1. Example Reads Excluded By Filters. **A)** Reads filtered out by genomic filter for mapping better to pseudogenes & segment duplicated regions **B)** Reads excluded by the transcriptomic filter for having 100 % alignment to a canonical splice between exons 10 and 11 of HNRNPH1 **C)** Reads excluded by applying the junctional filters, segment PID and JSpan (see text). (PDF 475 kb) Additional file 4: Table S2. circRNA transcripts published by Memczak et al. [4] with 100 % overlap to annotated pseudogenes and excluded by PTESFinder. (XLSX 12 kb)
