# Erratum to: PTESFinder: a computational method to identify post-transcriptional exon shuffling (PTES) events

**DOI:** 10.1186/s12859-016-0949-1

**Published:** 2016-02-18

**Authors:** Osagie G. Izuogu, Abd A. Alhasan, Hani M. Alafghani, Mauro Santibanez-Koref, David J. Elliott, Michael S. Jackson

**Affiliations:** 1 grid.1006.70000000104627212Institute of Genetic Medicine, Newcastle University, Newcastle Upon Tyne, UK; 2 Security Forces Hostpital, P. O. Box 2748-24268-8541, Makkah, Kingdom of Saudi Arabia

Upon publication of the original article [1] it has been found that author David J. Elliott has been accidentally misspelt. The correct spelling is David J. Elliott rather than David J. Elliot. This has been herewith corrected with this erratum and also updated in the original article.
