# Supplementary material for: PTESFinder: a computational method to identify post-transcriptional exon shuffling (PTES) events
Source: BMC Bioinformatics. 2016 Jan 13;17:31. doi: 10.1186/s12859-016-0881-4 (PMC4711006; doi:10.1186/s12859-016-0881-4)
Supplement: Additional file 2: Table S1. — Analyses of RNASEQ data from human fibroblast cells. (PDF 19 kb) [file 12859_2016_881_MOESM2_ESM.pdf]

SUPPLEMENTARY TABLE 1: ANALYSES OF RNASEQ DATA FROM HUMAN FIBROBLAST CELLS

	**SRR444974A (RNase R Digested)**	**SRR444975A (Undigested)**
Library Size	158305855	206362733
Reads Detected With Shuffled Coordinates - ***discovery phase***	471109	359837
Reads Mapped To PTES Models - ***evaluation phase***	129347	44620
Reads Excluded By Genomic Filter	9330	24195
Reads Excluded By Transcriptomic Filter	12213	30401
Excluded Reads with 100% Alignment to PTES	*[115]*	*[228]*
Reads Excluded By Both Genomic & Transcriptomic	*[6317]*	*[23778]*
Reads Excluded By Genomic Filter Only	*[3013]*	*[417]*
Reads Excluded By Transcriptomic Filter Only	*[5896]*	*[6623]*
Reads Excluded By Junctional Filter (PID=60, Jspan=4)	32623	34820
Reads Excluded By Junctional Filter Only (PID=60, Jspan=4)	26372	7036
