# Supplementary figures and images for: PTESFinder: a computational method to identify post-transcriptional exon shuffling (PTES) events
Source: BMC Bioinformatics. 2016 Jan 13;17:31. doi: 10.1186/s12859-016-0881-4 (PMC4711006; doi:10.1186/s12859-016-0881-4)

**A**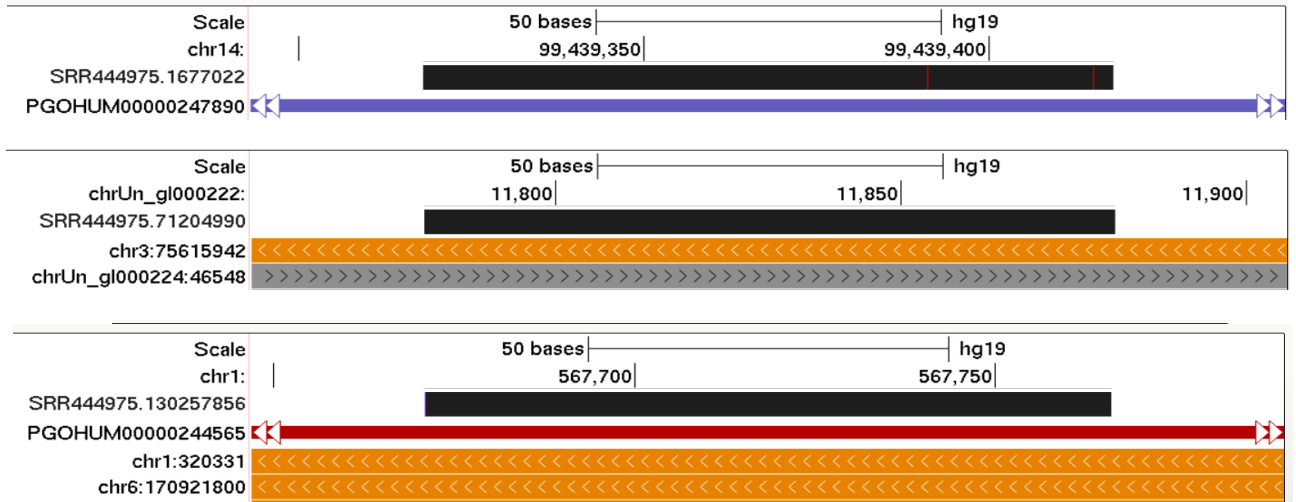**B**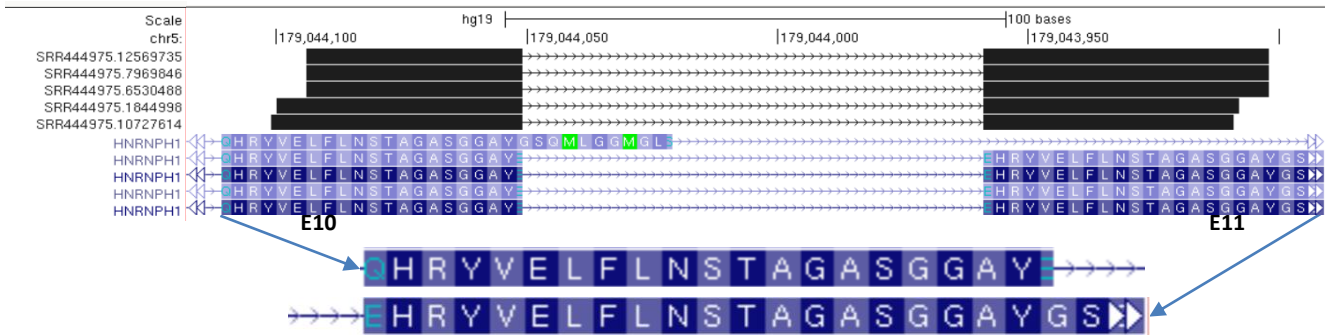**C**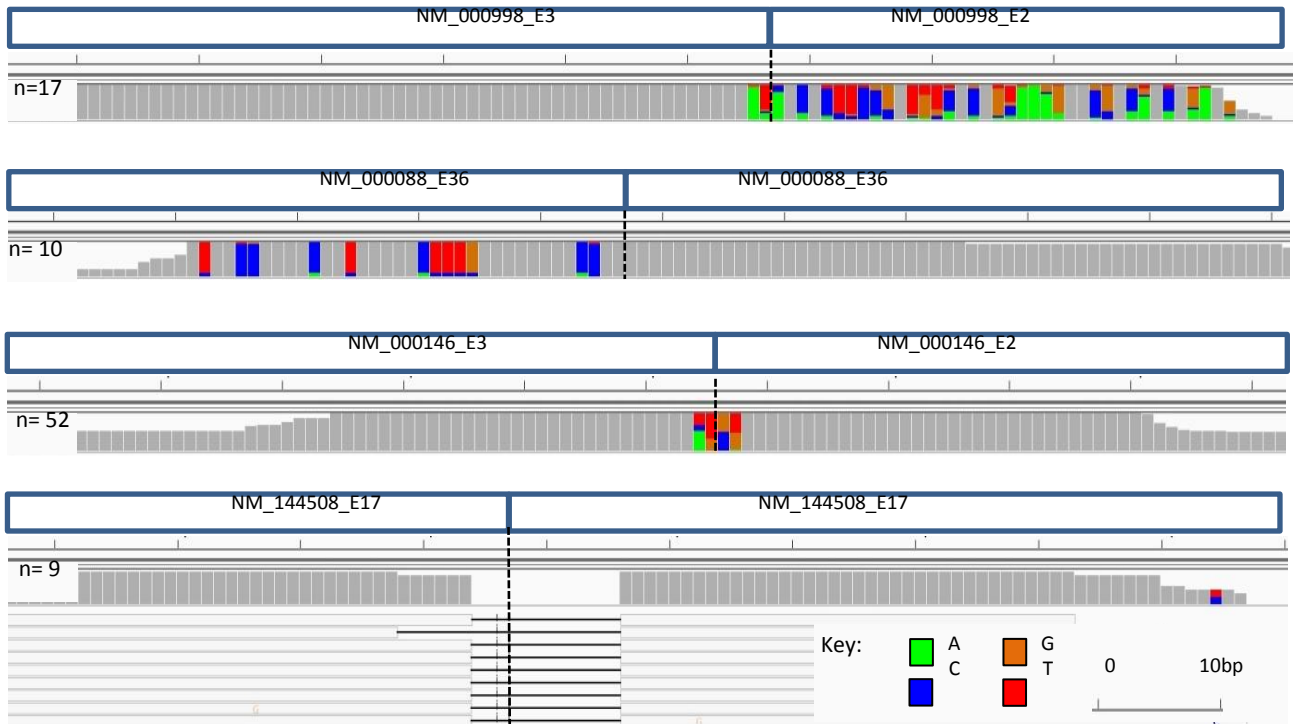

Supplement: Additional file 3: Figure S1. — Example Reads Excluded By Filters. A) Reads filtered out by genomic filter for mapping better to pseudogenes & segment duplicated regions B) Reads excluded by the transcriptomic filter for having 100 % alignment to a canonical splice between exons 10 and 11 of HNRNPH1 C) Reads excluded by applying the junctional filters, segment PID and JSpan (see text). (PDF 475 kb) [file 12859_2016_881_MOESM3_ESM.pdf]
